# Malaria parasite diversity and transmission intensity affect development of parasitological immunity in a mathematical model

**DOI:** 10.1186/1475-2875-11-419

**Published:** 2012-12-15

**Authors:** Philip A Eckhoff

**Affiliations:** 1 Intellectual Ventures Laboratory, 1600 132nd Ave NE, Bellevue, WA, 98004, USA

## Abstract

**Background:**

The development of parasitological immunity against malaria affects the ability to detect infection, the efficiency of the local human parasite reservoir at infecting mosquitoes, and the response to reintroduction of parasites to previously cleared areas. Observations of similar age-trends in detected prevalence and mean parasitaemia across more than an order-of-magnitude of variation in baseline transmission complicate simple exposure-driven explanations.

**Methods:**

Mathematical models often employ age-dependent immune factors to match the observed trends, while the present model uses a new detailed mechanistic model of parasite transmission dynamics to explain age-trends through the mechanism of parasite diversity. Illustrative simulations are performed for multiple field sites in Tanzania and Nigeria, and observed age-trends and seasonality in parasite prevalence are recreated *in silico*, proffering possible mechanistic explanations of the observational data.

**Results:**

Observed temporal dynamics in measured parasitaemia are recreated for each location and age-prevalence outputs are studied. Increasing population-level diversity in malaria surface antigens delays development of broad parasitological immunity. A local parasite population with high diversity can recreate the observed trends in age-prevalence across more than an order of magnitude of variation in transmission intensities.

**Conclusions:**

Mechanistic models of human immunity and parasite antigen diversity can recreate the observed temporal patterns for the development of parasitological immunity across a wide range of transmission intensities. This has implications for the distribution of disease burden across the population, the human transmission reservoir, design of elimination campaigns, and development and roll-out of potential vaccines.

## Background

Notable progress has been made against malaria over the past seven years, with the burden of malaria decreasing from an estimated 500 million cases and 1 million deaths in 2005 [1] to an estimated 200 million cases and 650,000 deaths in 2010 [2]. Despite this progress, the burden of malaria remains intolerably high, and current gains may be fragile. Interventions may wear out and require replacement or become less useful due to resistance. Malaria is thus the focus of a global effort aimed at its eradication.

Design of elimination campaigns should not follow a one-size-fits-all pattern due to geographic heterogeneity in malaria epidemiology. High levels of geographic heterogeneity exist in transmission intensity [3], local vector species [4], and population densities and housing conditions [5]. Not only is the epidemiology highly heterogeneous geographically, but it is changing rapidly through urbanization in Africa [6] and the vast scale-up in intervention coverage [2]. Success in the ongoing eradication campaign will not be due to a single intervention, but to combinations of interventions tailored to local circumstances. In some cases, attainment of local elimination may require development of new tools such as vaccines or novel means of vector control. The challenges facing malaria eradication define an ambitious research agenda as outlined by the MalERA framework [7,8].

Several key questions concern the development of immunity to severe disease, symptomatic disease, and the parasite itself due to previous exposure and infection. Prior exposure can influence the clinical outcome of a new infection [9], the extent of symptoms [10], and the probability of transmission [11,12], all of which have programmatic implications. Different time intervals are observed to be involved in acquisition of immunity to severe malaria, to symptomatic malaria, and to detectable malaria [13], but sterilizing immunity is not observed in natural conditions. Immunity to severe malaria can develop in a few years, but rates of detected parasitaemia tend to remain high until the age of ten and then decrease slowly towards adult levels beyond the age of twenty [14]. Malaria exhibits tremendous antigenic variation, both within single infections [15] and across the parasite population [16], and variant-specific exposure is a strong predictor of future responses [10]. Adaptive immunity is also observed to *Plasmodium* merozoites [17], and the innate inflammatory immune response is important for parasite control and patient symptoms [18]. These human immune responses and parasite antigenic diversity interact to create the patterns of immune acquisition, but modelling these dynamics can be difficult.

Mathematical modelling of malaria has a rich history dating back to the work of Ross and Macdonald and their contemporaries, which established key principles for the first global eradication campaign [19-21]. Mathematical modelling of malaria made major leaps forward through the Garki Project and the model built to study it [22], as well as modern models for population dynamics which followed [23-27]. In addition to population-level models, recent research has advanced the state of detailed models of the within-host dynamics of *Plasmodium falciparum*[28-30]. Models have been constructed to study antigenic variation at the single infection level [31,32]. Other work has advanced the state of modelling of vector population dynamics and intensity of vector-borne disease transmission [33-36]. An important new class of models combines population-level transmission with an ever richer representation of individual-level infection and immune dynamics [14,25,26,37,38].

Mathematical models have been used to understand the development of immunity over time and exposure at the population level [14,37]. These approaches tend to use combinations of age, integrated exposure, or total infections to set overall immune levels as a proxy for the specific-immune responses acquired over time. The OpenMalaria model [14,25], for example, implements pre-erythrocytic immunity, maternal antibodies, changes in biting exposure with age, cumulative number of infections, and lifetime integrated parasite density to achieve good fits to age-prevalence data without distinguishing among the specific antigens for each infection. Multiple other models also use combinations of age and antigen-agnostic exposure to recreate the acquisition of immunity [22,26,37]. At another end of the modelling spectrum, models based on strain theory [39] tend to represent acquisition of immunity as the gradual development of quasi-sterilizing immunity to a large repertoire of separate strains with distinct antigenic profiles for each infection. The lack of observed sterilizing immunity to primary infection is one of multiple issues with strain theory, but the observed roles of immune response to specific antigens [10] and antigen-specific acquisition of immunity [41] illustrate possible shortcomings of antigen-agnostic models that rely only on age and cumulative exposure.

This work presents a new model which combines previous work on individual-based models that contain detailed local vector population dynamics [42] with a more detailed representation of within-host parasite dynamics and immune responses [43]. It aims to develop and exercise a detailed model of transmission with a parasite population exhibiting multiple forms of clonal and intraclonal antigenic variation to which individual human agents in the simulation acquire immunity over the course of repeated exposures. Each new simulated infection may have a mix of previously-experienced and novel antigens. High transient immune responses eventually clear ongoing infections when the parasite fails to introduce an antigenic variant which can propagate. After infections are cleared, antibody responses decay to lower levels of specific immunological memory. Immunological memory reduces the parasite density of future infections presenting that antigen and may aid in the eventual clearance of such infections, although sterilizing immunity is not attained.

The present model is used to investigate and illustrate questions about the acquisition of immunity and its dependence on age and exposure. The acquisition of immunity as a function of age and exposure is compared to field data from the Garki Project [22,44] and other field sites in Namawala, Tanzania [45]. The diversity in parasite antigens across the entire parasite population in a region affects the time for the development of parasitological immunity and the population dynamics. By implementing a repertoire of specific antigens to which non-sterilizing immunity is acquired over time, the present model provides a platform for studying the roles of antigen-specific immunity and diversity in the parasite population without introducing less-substantiated assumptions of strain theory. As such, this platform can be used to explore more mechanistic interpretations of well-established relationships of immunity with age and exposure.

## Methods

### Model structure overview

The core of the simulation consists of solvers for mosquito dynamics, transmission to and from the human host population, and dynamics of the infection within the human host. The model for mosquito life cycle and feeding is described in full detail elsewhere but is summarized below [42]. While the mosquito population can be represented by discrete cohorts or individual agent mosquitoes, the human population is fully individual-based. The mosquito population is advanced through discrete 1-day time steps between night-time intervals of feeding on the human population, during which susceptible mosquitoes can become infected and individual humans can receive new infections. Each *Plasmodium falciparum* infection in an individual human is represented by a parasite dynamics microsolver, described in detail in [43]. The model for parasite dynamics and immune response uses a series of discrete and continuous process to achieve more mechanistic representations of underlying processes. Discrete events such as the ending of an asexual cycle, the associated rupturing of schizonts, and the creation of a new generation of infected red blood cells are governed by a timer whose expiration triggers these discrete events. Between such events, development of the immune response and clearance of infected red blood cells are represented by continuous differential equation processes approximated with a one-hour time step. Parameters and variables are summarized in Table 1, and further details and literature sources can be found in the articles for each component [42,43]. Each component of the model is now explained in detail.

**Table 1:** Parameters and variables

**Parameter name**	**Value in simulations**	**Variable name**	**Quantity measured**
***n***_***PfEMP1***_	300-1000	***t***_***asexual***_	Time remaining in current asexual cycle
***n***_***minor***_	20-100	***N***_***i***_	Number of IRBCs of type i
***n***_***MSP***_	4-100	***X***_***i***_	Density of IRBCs of type i per μL
***X***_***50,innate***_	1000/μL	***Y***_***capacity,f(i)***_	Non-dimensional capacity to mount antibody response to variant f(i)
***τ***_***innate***_	0.5 days	***Y***_***antibody,f(i)***_	Non-dimensional antibody response to variant f(i)
***k***_***fever***_	2 C	***Y***_***innate***_	Non-dimensional level of inflammatory cytokines
***X***_***50,antibody***_	10/μL	***Y***_***fever***_	Fever in degrees C
***τ***_***capacity***_	10 days	***Y***_***MSP,j***_	Adaptive immune response to MSP variant j
***k***_***antibodymin***_	0.01	***N***_***i+j,i***_	Number of IRBCs of type i with progeny expressing type i + j
***k***_***minormod***_	0.5	***Z***_***MS***_	Success probability of merozoite invasion
***C***_***innate***_	0.2	***N***_***gametocytes,i***_	Number of gametocytes of stage i
***C***_***antibody***_	1.5	***X***_***gametocytes,mature***_	Mature female gametocytes per μL
***c***_***minormod***_	0.3	***P***_***kill,i***_	Probability of clearing IRBC of type i
***τ***_***abdecay***_	20 days	***P***_***infect***_	Probability of successfully infecting mosquito
***Y***_***memory***_	Varies up to 0.3		
***K***_***MSP***_	0.02		
***K***_***antigen***_	5e-9		
***n***_***antigenswitch***_	7		
***k***_***gametocyte***_	0.1		
***C***_***merozoite***_	0.5		
***N***_***IRBCmerozoites***_	16		
***T***_***asexual***_	2 days		
***Z***_***success***_	0.01		
***Y***_***gam,50***_	0.05		

### Mosquito dynamics

Multiple *Anopheline* species can be tracked simultaneously, each with potentially different behaviors and ecologies, and thus possibly responding distinctly to different combinations of interventions. Within a single species population, adult mosquitoes can be represented as individual agents in the simulation or grouped as identical-state cohorts. The number of required cohorts corresponds to the number of occupied unique states for mosquitoes, and more detail and complexity requires more unique states. The individual-agent version of the vector population model scales with the number of local vectors at a given time.

Mosquitoes begin the life cycle as eggs oviposited by existing adult females following successful feeds. Following hatching, cohorts progress through larval and pupal development at temperature-dependent rates to emergence. Progress through this phase is tracked by a cohort-specific variable which increases from zero by a temperature-dependent amount each time step until it reaches completion, and thus this representation allows varying temperatures and varying lengths of larval development in a simple and memory-sparing structure. All mosquitoes in the cohort are assumed to experience the same temperatures on a given time step. Following emergence, there is a brief delay during which sugar feeding and mating occur. Following mating, the adult females begin a cycle of feeding and oviposition. Feeds upon gametocyte-carrying humans carry a chance of infection, and mosquitoes that are successfully infected by the parasite change state to infected and progress at a temperature-dependent rate towards infectiousness. The structure and implementation of the progress variable is similar to the implementation for larval progress, but with a different temperature-dependent rate function. Successful blood meals lead to oviposition, and this closed-loop egg laying can thus capture the local elimination of certain species following intense vector control.

### Within-host parasite dynamics

These vector population and transmission models are coupled to microsolvers for the natural history of the parasite within a human host [43]. The vector population and transmission models are simulated with a one-day time step, to correspond to the interval between nights of vectors feeding. The intrahost microsolver uses a combination of discrete and continuous processes to represent parasite dynamics and immune responses [43]. The parasite begins an infection in the human liver and emerges after a latency of seven days. Each infected red blood cell (IRBC) expresses one *Plasmodium falciparum* Erythrocyte Membrane Protein 1 (PfEMP-1) surface antigen out of 50 for that infection and one minor epitope out of a set of five. The 50 PfEMP-1 antigens are drawn from the overall population of possible antigens *n*_*PfEMP1*_ (varying between 300 and 1,000 in the present results), as is done for each of the sets of five minor epitopes *n*_*minor*_ (varying from 20 to 100 total minor epitopes) and for the merozoite surface antigen *n*_*MSP*_ (varying from 4 to 100 in the present results). An indexing function *f*_*j*_*(i*) relates PfEMP-1 antigen i in infection j to the overall list of antigens which extends from 1 to *n*_*PfEMP1*_. A related indexing function maps which minor epitope in *n*_*minor*_ corresponds to the specific variant i. Superinfection is supported in this model, and each concurrent infection will be simulated individually with its own set of indexing functions. These infections all interact with the same immune system at the same time, and the immune microsolver governs the dynamics of responses to all antigens, not just the antigens of a single infection.

#### Asexual cycle parasite dynamics and immune response

The asexual cycle is a discrete two-day cycle governed by a timer *t*_*asexual*_, with each IRBC releasing 16 merozoites approximately 48 hours after its invasion by an earlier generation of merozoites. During these two day intervals, the infection is simulated with a one-hour time step Δt, with the following equations tracking the numbers of IRBCs of each antigen type *N*_*i*_, the density of IRBCs of each type per μL *X*_*i*_, the capacity to produce antibodies specific to a given antigen *Y*_*capacity*,*f*(*i*)_, the antibody concentrations *Y*_*antibody*,*f*(*i*)_, and the general inflammatory response *Y*_*innate*_ and fever *Y*_*fever*_.

(1) $$t_{\mathrm{asexual},n+1}=t_{\mathrm{asexual},n}-\mathrm{\Delta t}$$

(2) $$\frac{dY_{\mathrm{innate}}}{\mathrm{dt}}=\frac{1}{\tau_{\mathrm{innate}}}\frac{\sum_{i}X_{i}\left(1-Y_{\mathrm{antibody},i}\right)}{\sum_{i}X_{i}\left(1-Y_{\mathrm{antibody},i}\right)+X_{50,\mathrm{innate}}}$$

(3) $$Y_{\mathrm{fever}}=k_{\mathrm{fever}}Y_{\mathrm{innate}}$$

For i = 1 to 50, if *X*_*i*_ > 0,

(4) $$\begin{array}{l} \frac{dY_{\mathrm{capacity},\mathrm{fj}\left(i\right)}}{\mathrm{dt}}=\frac{1}{\tau_{\mathrm{capacity}}}\frac{X_{i}+k_{\mathrm{antibody}\min}X_{50,\mathrm{antibody}}}{X_{i}+X_{50,\mathrm{antibody}}}\\ \;\times \left(1-Y_{\mathrm{capacity},\mathrm{fj}\left(i\right)}\right),\\ \;Y_{\mathrm{capacity},\mathrm{fj}\left(i\right)}<0.4,\;\mathrm{PfEMP}-1 \end{array}$$

(5) $$\begin{array}{l} \frac{dY_{\mathrm{capacity},\mathrm{fj}\left(i\right)}}{\mathrm{dt}}=\frac{k_{\min\;\mathrm{or}\mathrm{mod}}}{\tau_{\mathrm{capacity}}}\frac{X_{i}+k_{\mathrm{antibody}\min}X_{50,\mathrm{antibody}}}{X_{i}+X_{50,\mathrm{antibody}}}\\ \;\times \left(1-Y_{\mathrm{capacity},\mathrm{fj}\left(i\right)}\right),\\ \;Y_{\mathrm{capacity},\mathrm{fj}\left(i\right)}<0.4,\;\mathrm{minor}\;\mathrm{epitopes} \end{array}$$

(6) $$\begin{array}{l} \frac{dY_{\mathrm{capacity},\mathrm{fj}\left(i\right)}}{\mathrm{dt}}=\frac{1}{\tau_{\mathrm{hyperimmunity}}}\left(1-Y_{\mathrm{capacity},\mathrm{fj}\left(i\right)}\right),\\ \;Y_{\mathrm{capacity},\mathrm{fj}\left(i\right)}\geq 0.4 \end{array}$$

(7) $$\begin{array}{l} \frac{dY_{\mathrm{antibody},\mathrm{fj}\left(i\right)}}{\mathrm{dt}}=\frac{1}{\tau_{\mathrm{abprod}}}\left(Y_{\mathrm{capacity},\mathrm{fj}\left(i\right)}-Y_{\mathrm{antibody},\mathrm{fj}\left(i\right)}\right),\\ \;Y_{\mathrm{capacity},\mathrm{fj}\left(i\right)}>0.3 \end{array}$$

(8) $$P_{\mathrm{kill},i}=1-\exp\left(-\mathrm{\Delta t}\left(\begin{array}{l} C_{\mathrm{innate}}\frac{Y_{\mathrm{fever}}}{1+Y_{\mathrm{fever}}}+C_{\mathrm{antibody}}\\ \left(Y_{\mathrm{antibody},\mathrm{fj}\left(i\right)}+Y_{\mathrm{Minorepitope},\mathrm{fj}\left(i\right)}c_{\mathrm{MinorMod}}\right) \end{array}\right)\right)$$

(9) $$\begin{array}{l} N_{i}^{n+1}=N_{i}^{n}-\mathrm{Binomial}\left(N_{i}^{n},P_{\mathrm{kill},i}\right)\;\mathrm{the}\;n\;\mathrm{and}\\ \;n+1\;\mathrm{indicate}\;\mathrm{succesive}\;\mathrm{time}\;\mathrm{steps} \end{array}$$

If no IRBCs for any current infection are expressing a given antigen, then antibody levels corresponding to that antigen will decline, and capacity to produce that specific antibody will decay towards a memory level *Y*_*memory*_. That is, for i = 1 to *n*_*PfEMP1*_, if Σ *X*_*fj*-*1*(*i*)_ = 0, in which *f*_*j*_^-*1*^(*i*) is the inverse indexing for each of the current infections 1 to j,

(10) $$\begin{array}{l} \frac{dY_{\mathrm{capacity},i}}{\mathrm{dt}}=-\frac{1}{\tau_{\mathrm{capdecay}}}\left(Y_{\mathrm{capacity},i}-Y_{\mathrm{memory}}\right),Y_{\mathrm{capacity},i}\\ \;>Y_{\mathrm{memory}} \end{array}$$

(11) $$\frac{dY_{\mathrm{antibody},i}}{\mathrm{dt}}=-\frac{1}{\tau_{\mathrm{abdecay}}}Y_{\mathrm{antibody},i}$$

Once the asexual cycle timer runs out, schizonts rupture, merozoites are released, and a new cycle’s IRBCs are created. There is an additional inflammatory stimulation due to rupturing schizonts and the release of haemozoin and other byproducts.

(12) $$\Delta Y_{\mathrm{innate}}=\frac{\sum X_{\mathrm{schizont}}}{\sum X_{\mathrm{schizont}}+X_{50,\mathrm{innate}}}$$

Merozoite-specific immunity is also stimulated for the merozoite surface antigen expressed by each current infection 1 to j as this is the brief interval in which merozoites are free in the bloodstream.

(13) $$\Delta Y_{\mathrm{MSP},j}=K_{\mathrm{MSP}}\frac{\sum X_{\mathrm{schizont},j}}{\sum X_{\mathrm{schizont},j}+X_{50,\mathrm{antibody}}}\left(1-Y_{\mathrm{MSP}.j}\right)$$

A simplified model for antigenic switching is implemented, and the numbers of parasites expressing each PfEMP-1 variant are tracked. The rate of antigenic switching is per IRBC, so more new variants are introduced at high parasite densities. The number of IRBCs of antigen n switching to express antigen i + j is calculated as

(14) $$N_{i+j,i}=\mathrm{Poisson}\left(K_{\mathrm{antigen}}N_{i}\right),1\leq j\leq n_{\mathrm{antigenswitch}},$$

which leaves the number of IRBCs of antigen n created by previous generation expressing antigen n as

(15) $$N_{i}^{1}=\left(\left(1-k_{\mathrm{gametocyte}}\right)N_{i}-\sum_{j=1}^{\mathrm{nantigenswitch}}N_{i+j,i}\right)N_{\mathrm{IRBCmerozoites}}Z_{\mathrm{MS}}$$

To get the total number of new IRBC’s in the next generation of a given expressed antigen, for all i, j = 1 to n_antigenswtich_, add in the switching IRBCs.

(16) $$N_{i+j}^{1}+=N_{i+j,i}N_{\mathrm{IRBCmerozoites}}Z_{\mathrm{MS}}$$

The quantity *Z*_*MS*_ refers to the success probability of merozoite invasion, which is reduced as merozoite specific antibody responses increase and is calculated as

(17) $$Z_{\mathrm{MS}}=\left(1-C_{\mathrm{merozoite}}Y_{\mathrm{antibody},\mathrm{MSP}}\right)$$

#### Gametocyte production and transmission

Some IRBCs produce male or female gametocytes, which go through a ten-day development process to maturity, moving forward a stage every asexual cycle.

(18) $$N_{\mathrm{gametocyte},i}=N_{\mathrm{gametocyte},i-1},i=5:1$$

(19) $$N_{\mathrm{gametocytes},0}=\sum_{i}N_{i}k_{\mathrm{gametocyte}}N_{\mathrm{IRBCmerozoites}}Z_{\mathrm{MS}}$$

Finally, after all the updates at the end of a 2-day cycle have been processed, the asexual timer is reset for the next generation and the simulation continues on a 1-hour timestep until the timer is completed again.

(20) $$t_{\mathrm{asexual}}=T_{\mathrm{asexual}}.$$

The concentration of mature female gametocytes per μL, *X*_*gametocytes*,*mature*_, is used to calculate infectiousness to the mosquito of a 2 μL bloodmeal. This is combined with a basic rate of success per gametocyte *Z*_*success*_ and a sigmoidal function of the inflammatory response to represent the decreased infectiousness of gametocytes incubated with inflammatory cytokines [46].

(21) Pinfect=1−e^−2Xgametocytes,matureZsuccess1−YinnateYgam,50+Yinnate

#### Immune memory

The human immune response within the microsolvers described by the above equations consists of a broad innate inflammatory response as well as specific antibody responses for each antigenic variant of each type. Increasing concentrations of novel antigens stimulate both an inflammatory response and a delayed antibody response. As the antibody response increases on a dimensionless scale from 0 to 1, the initial inflammatory response is suppressed. After the antibody response appears, continued antigenic stimulation will drive up the adapted response until that antigenic variant is cleared, at which point both the antibody levels and the capacity to respond will decay over time. The capacity to generate specific antibodies upon challenge can be set to decay to a nonzero value *Y*_*memory*_, allowing a shorter latency to response upon reinfection. Higher values of *Y*_*memory*_ yield stronger immune response and lower rates of detected parasitaemia upon reinfection, and this results in lower parasite densities in the adult human population. The larger the antigenic population, the more infections and thus time it takes to acquire broad parasitological immunity.

## Results

**Figure 1:**
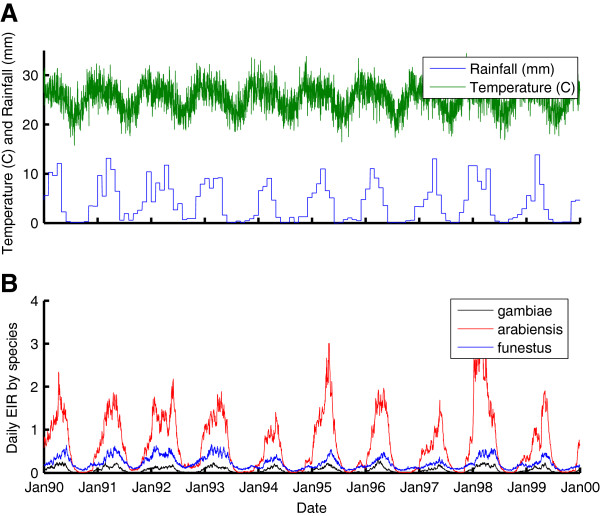
**Creation of Namawala simulation baseline. A**) Historical temperature and rainfall. **B**) Simulated daily EIR by vector species after fitting to mix of species and transmission intensity.

The basic simulation for Namawala, Tanzania in the early 1990’s [45] is constructed as described in [42]. The rainfall for January 1990 through December 1999 was obtained from GPCC [47], and the temperatures from Swiss TPH [25,45]. The simulation includes distinct local populations of *Anopheles gambiae s*.*s*, *Anopheles arabiensis*, and *Anopheles funestus*, and the available habitat factor for each species is set through fitting simulations of the basic vector model running with specified weather to EIR data per species. The results are displayed in Figure 1, with an average annual EIR of 244 due to *An*. *arabiensis*, 25 due to *An*. *gambiae s*.*s*, and 84 due to *An*. *funestus*. The *An*. *funestus* component rises and peaks towards the end of the rainy season, while the *An*. *gambiae s*.*l* component rises faster at the start of the rainfall. The substantial year-to-year variability has a stochastic component, but is primarily driven by year-to-year differences in rainfall intensity and patterns. The same temperature pattern based on averages for 1991–1993 was used for each year, and using the actual daily temperatures for the decade would cause further year-to-year variability.

**Figure 2:**
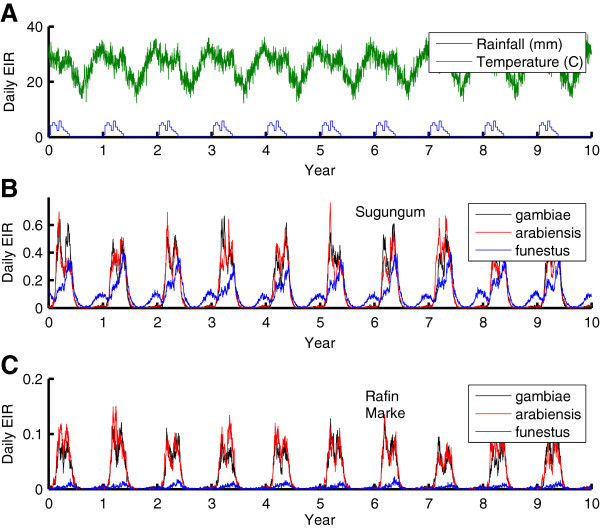
**Creation of Garki district simulation baseline. A**) Historical temperature and rainfall. **B**) Simulated daily EIR by vector species for village of Sugungum. **C**) Simulated daily EIR by vector species for village of Rafin Marke.

In addition to Namawala, simulations are constructed for various villages from the Garki District in Nigeria that was the focus of an intense, well-instrumented anti-malaria campaign in the early 1970s [22]. Rainfall, humidity, and air temperatures were recorded for the district and are used to drive the present simulation. Entomological data were collected for various villages, tracking *An*. *funestus* and *An*. *gambiae s*.*l*. *Anopheles gambiae s*.*s* and *An*. *arabiensis* were not distinguished, but *An*. *gambiae s*.*l* was observed to rest indoors only 47 percent of the time. The recorded entomological and weather data are used to construct local simulations for the villages of Sugungum and Rafin Marke, which had the highest and lowest EIR of the study area villages, respectively. In addition, the vector population around Sugungum exhibited a substantial *An*. *funestus* component, which extends transmission further into the dry season than *An*. *gambiae s*.*l*, unlike Rafin Marke. The weather for the Garki District during the Garki project and the results of baseline simulations for Sugungum and Rafin Marke can be seen in Figure 2.

The development of anti-parasite immunity over age and exposure can be modelled with functions of age and exposure [37], or with exposure and infections with an age parameter in biting exposure [14]. Part of the challenge facing mathematical models is that over a wide range of transmission intensities there is a high level of detected parasitaemia through the age of 10 followed by a decline to a lower level after the age of 20 [14,22,45]. The total exposure experienced varies by over an order of magnitude, and yet the development of immunity limiting the rate of detection of parasite infection varies much less. These dynamics can be fit by incorporating a strong age effect in the immune functions [37], but it is unclear whether such a strong mechanism with these age-linked characteristics exists. In addition, many existing models treat every infection the same, and ignore antigenic diversity across clones.

**Figure 3:**
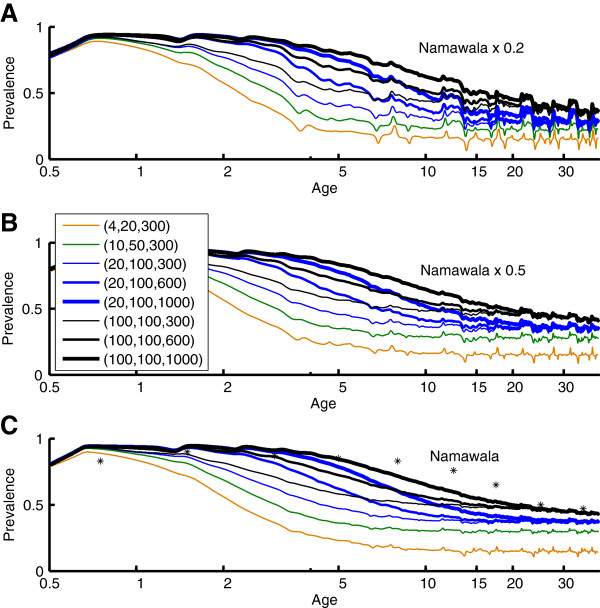
**Age-prevalence curves for Namawala transmission scaled by A) 0.2, B) 0.5, and C) 1.0 times baseline vector dynamics.** A separate age-prevalence trajectory is displayed for each parasite population diversity combination of (merozoite variants, sets of minor epitopes, PfEMP-1 variants). Parasite diversities with the same number of merozoite variants and the same number of sets of minor epitopes asymptote to the same prevalence, but parasite diversities with greater numbers of PfEMP-1 variants take longer to converge. Parasite densities and prevalence values are smoothed with a 1-year window. Actual data for Namawala is overlaid in panel C.

**Figure 4:**
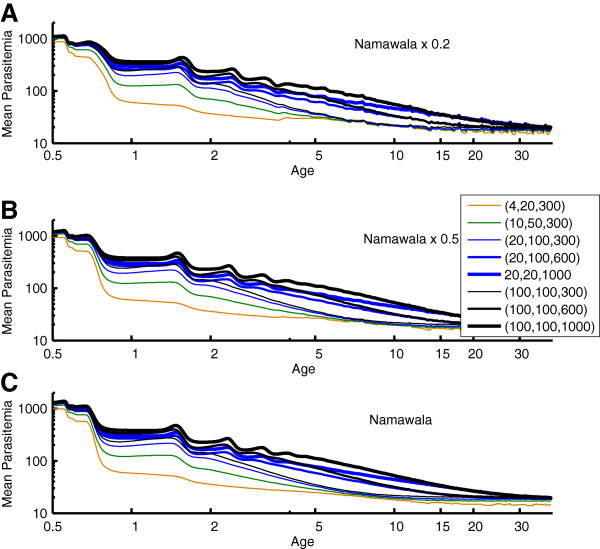
**The mean parasitaemia by age corresponding to the Namawala simulations in Figure**3. Mean parasitaemia decreases from a maximum of approximately 1000 parasites/μL to an asymptote less than 100 parasites/μL as observed in field data.

**Figure 5:**
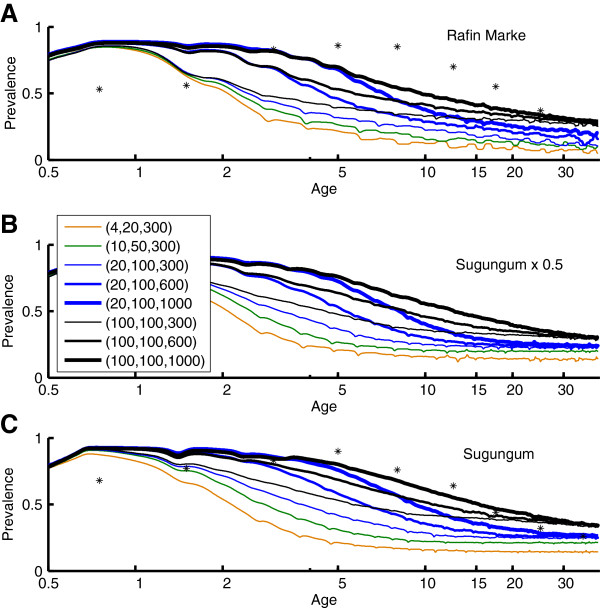
**Age-prevalence for Garki seasonality and baseline vector populations corresponding to target EIRs of A) ~24, B) ~60, and C) ~120.** Observed values for Rafin Marke and Sugungum are overlaid on panels A and C, respectively. Parasite population diversity is as in Figure 3.

**Figure 6:**
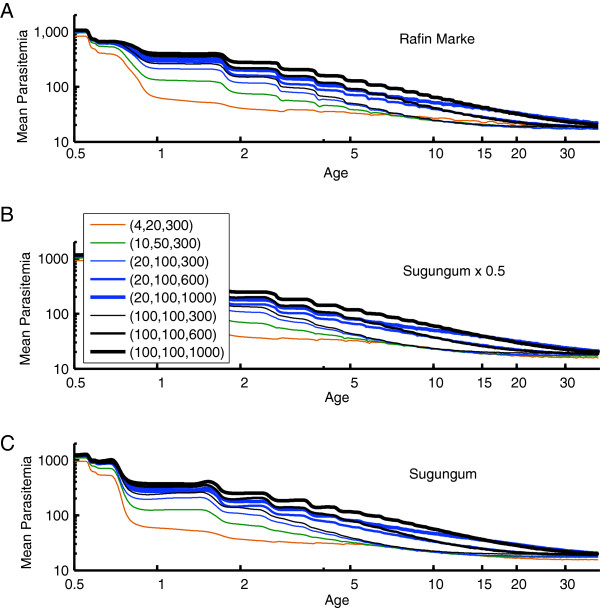
**Mean parasitaemia by age corresponding to the Garki-based simulations in Figure**5.

It has been proposed that antigenic diversity in the local parasite population plays a role in this development of immunity [10]. The present simulations implement a local population with a specified number of merozoite-surface antigens [17,48], PfEMP-1 variants [10,49], and minor antigenic epitopes associated with infected red blood cells [31]. Specific clones are not tracked through the vector population in the present results, although the model structures would support such tracking. Each new human infection draws its antigenic repertoire from the total population set. The resulting strain structure is different from traditional strain theory in that these sets of antigens can partially overlap, and immunity to an infection is not sterilizing even if all antigens are repeated. Figures 3, 4, 5, and 6 show the results of measured prevalence and parasite density by age for populations of 1) 4 merozoite, 20 minor epitopes, 300 PfEMP-1 variants, 2) 10 merozoite, 50 minor epitopes, 300 PfEMP-1 variants, 3) 20 merozoite, 100 minor epitopes, and 300, 600 or 1,000 PfEMP-1 variants, and 4) 100 merozoite, 100 minor epitopes, and 300, 600 or 1,000 PfEMP-1 variants, referenced below as (merozoite, minor epitopes, PfEMP-1). In these simulations, as the population ages, the transmission levels do moderately decline, but this does not substantially change the trends in detected prevalence or parasite densities.

Figures 3 and 4 show the change in detected parasite prevalence and geometric mean parasitaemia smoothed with a one-year window for Namawala seasonality and either baseline vector populations, 0.5 baseline, or 0.2 baseline. The “0.5 x Namawala” simulation exhibits approximately half of the adult vectors per person as experienced in the baseline Namawala simulation. The actual EIR can vary depending on the parasite reservoir in the human population. The number of available merozoite and minor epitope combinations sets the asymptote for older age groups, and increasing the number of PfEMP-1 variants in the population increases the time for approaching this asymptote. All of the (100, 100, N) trajectories end with the same prevalence, but the one with N = 1,000 PfEMP-1 variants takes much longer to reach this level and begins to approach patterns seen in the Namawala data [14], which is displayed in panel 3C as point data. Simulations with too little parasite population diversity, for example (4, 20, 300), reach age-prevalence equilibrium after just 3–5 years, which is not realistic. Increasing the number of local parasite antigenic variants drives the age-prevalence curves to converge towards the actual data. This provides a simple, parsimonious, and mechanistic explanation for these curves.

Figures 5 and 6 repeat the analysis for Garki weather and EIRs ranging from a value characteristic of Rafin Marke (panel A) to one characteristic of Sugungum (panel C). Mean detected prevalence by age is plotted for Rafin Marke (panel A) and Sugungum (panel C) for comparison [14,22], using a detection sensitivity of 10 parasites per microliter [22]. Mean parasitaemia decreases from approximately 1,000 to below 100 parasites per microliter, as seen in the parasitological data [14,22]. Immune memory levels were set to 0.3, which represents high levels of immune memory within the low range of memory levels permitting observed reinfection dynamics, but such an immune memory with high parasite diversity produces an excellent approximation of observed patterns. With the (4, 20, 300) setting, an immune memory level of 0.225 can recreate the prevalence asymptote seen in field studies, but prevalence by age approaches the adult asymptote with a time-constant under four years. In addition, with a lower memory level of 0.225 the higher parasite diversity simulations asymptote at unrealistically high levels of detected parasitaemia. Thus the higher memory and diversity is preferred.

In addition to the temporally smoothed results for parasite prevalence and parasite density averaged by age, it is important to examine the temporal dynamics over the course of a year by age group. The magnitude of oscillations in detected prevalence over the year can change with transmission intensity, depth of the dry season, and age of the cohort. As seen in Figure 1, transmission is highly seasonal in Namawala, and yet field studies showed remarkably low seasonal variation in detected parasitaemia [45]. In Figure seventy-eight of the Garki Project report [22], temporal outputs from the Garki model are compared to the temporal pattern exhibited for each age range in the villages of Sugungum (highest transmission) and Rafin Marke (lowest transmission). Not much seasonality is observed for the younger age groups in Sugungum (although the 1–4 age group oscillates from 0.75 to 0.97 in Rafin Marke), but in the older age groups, a strong seasonal oscillation develops in detected parasitaemia. In Sugungum, in the 15–24 year age group, the low in April is 0.35 and the high in November is 0.55. In the 25–44 age group, the April low is 0.25 and the high of 0.45 was observed in August. In Rafin Marke, the 15–24 year age group has an oscillation from 0.38 in March to 0.8 in December, a dramatically high magnitude oscillation. The 25–44 age group ranges from 0.18 in March to 0.5 in October, a degree of oscillation underestimated by models with slower decay constants, including the Garki model itself [22].

**Figure 7:**
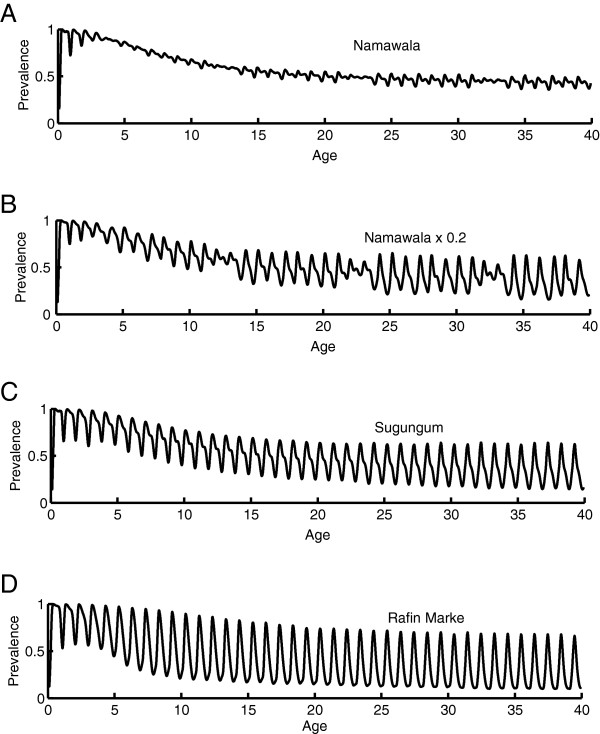
**Seasonal dynamics of detected parasitaemia by age for A) Namawala, B) Namawala with vector populations scaled down by a factor of 5, C) Garki with EIR of approximately 120, and D) Garki with an EIR of approximately 24.** For Namawala, the field-observed low seasonality in parasite dynamics is recreated, but seasonality begins to emerge when transmission is scaled down. For Garki, the adult oscillations approximately match those observed during the Garki Project, with D) Rafin Marke exhibiting a lower trough and higher peak than C) Sugungum.

The present model with parasite diversity recreates the changes with age in the temporal oscillations in detected parasitaemia as seen in Figure 7. Panel A shows the temporal dynamics for Namawala. Despite the strong level of seasonality, there is minimal fluctuation in detected parasitaemia, as observed in field studies [45]. When the EIR is reduced by a factor of 5, larger seasonal oscillations appear as seen in 7B. This can be explained by the fact that although the low season monthly EIR can be up to a factor of 50 below the highest observed months, most low-season months tend to have cumulative EIRs of at least six infectious bites per person. This maintains a steady supply of new infections. Dropping the vector population by a factor of 5 reduces the low season EIRs to levels that permit more substantial drops in detected parasitaemia. The simulations for the two Garki settings exhibit too high a magnitude of oscillation for the under 15-year olds, but the dynamics in the adult population exhibit an approximately correct range of oscillation. As in the field data, Rafin Marke, despite an annual EIR more than a factor of six below Sugungum’s, exhibits a higher peak and a lower trough. The trough can be explained by the extremely low dry-season EIR in Rafin Marke. Rafin Marke has a higher peak in rainy season prevalence in older age groups than Sugungum despite its lower transmission intensity. This feature is recreated in the model and is partially due to the lower dry-season parasitaemia leaving antibody titres lower to shared antigens going into the next high season.

## Discussion

The simulations presented in the foregoing demonstrate that parasite population diversity together with non-sterilizing immunological memory can explain the development of parasitological immunity with minimal *ad hoc* assumptions. The current model does not have an increase in biting rate exposure as age and body surface area increase, and thus modelled parasite prevalences for infants under one year of age are higher than observed, but incorporating age-dependent biting and maternal immunity as has been done elsewhere [14,50] would effectively shift the Rafin Marke, Sugungum, and Namawala curves rightward for the first year and produce an even better match to observations than reported above. Introduction of new parasite variants to the local population over time rather than all at the start of the simulation would extend the development of immunity to older age groups as well. These features were left out of present simulations to illustrate what can be achieved merely through the mechanism of immunity to an ever-growing repertoire of specific antigens. The role of such model platforms is not to produce a single best-fit set of parameters but to provide a simple and transparent way to understand the roles of specific biological and physiological mechanisms. The observed shift in age-prevalence curves with increasing parasite population diversity provides a mechanistic bridge between current antigen-agnostic models [14,22,26,34] and detailed antigen-specific field studies [10,17,41,51,52].

The presented simulations provide a simple and mechanistic explanation for a variety of field observations. First of all, the age-prevalence curve for Rafin Marke achieves the field-observed peak prevalence, although this peak is attained at too early an age. In addition, the convergence in the twenty years and older age group is an excellent match. In contrast, previous models tend to have too low a peak age-prevalence for Rafin Marke and too slow a decline to the old-age asymptote [14]. The present results achieve a more accurate peak and convergence to old-age asymptotic behaviour for Sugungum as well. The results for both villages have better matches on a linear age scale than many pre-existing models. The present simulations fall short on the under-one year old age group, but incorporation of maternal antibodies and decreased biting rates would resolve this issue. These features are left out to provide a simpler illustration of the acquisition of immunity to an antigenic repertoire over time. Finally, the presented simulations perform very well on temporal dynamics. The high oscillation in detected parasitaemia in older age groups in Garki villages is matched more accurately than by the Garki model itself [22]. And the present model provides a clear mechanistic interpretation of the low levels of seasonal variability in detected parasitaemia in Namawala as observed [45] and predicts an increase in seasonal variability as transmission levels decrease.

It is important to note that the model was not fitted to the parasitological data presented: the intrahost dynamics were parameterized from malariatherapy data and laboratory studies [43], and the vector population dynamics were constrained by entomological data [22,45]. Thus, although even better fits could be achieved by allowing all parameters to vary, the goal of the present work is to fix parameters that are better understood from clinical research datasets and then to discern the effects of less understood parameters. The effects of parasite population size are compared to field data which were not used to generate or fit the model. The success of parasite diversity in explaining age-prevalence patterns does not rule out age-dependent effects, and models which use age factors as a proxy for immunity will work very well and will be substantially computationally faster. The present model has a relatively high dimensionality and a correspondingly slow computational rate which make it suboptimal for some approaches, although the level of biomedical detail it admits supports study of questions that are difficult to examine in simpler frameworks.

The present model provides a useful mechanistic framework for recreating and comparing field observations of antibody profiles and responses. In general, the repertoire of antibody responses to PfEMP-1 variants increases with age in malaria endemic regions [41], and clinical disease tends to correspond to gaps in the antibody repertoire [10]. The role of transmission in the acquisition of this immunity can be seen in the similarity of specific antibody profiles in young children from the same region, having been exposed to contemporaneous parasites [41]. The present model incorporates known mechanisms such as immunological memory, which is observed for malaria antigens [51], while having antibody responses that decrease rapidly from hyper-immune levels post infection and exhibit longer-term decay on faster time scales than measles [51]. The power of these model structures is that they can examine these effects and implement known antigen-specific concepts and thus provide a mechanistic explanation for age-prevalence patterns of existing antigen-agnostic models.

Mechanistic models of vector dynamics can be exercised to study how sensitivities of local vector population dynamics to weather and other environmental variables can drive sensitivities to weather in parasite dynamics measured at the human population level. Environmental parameters such as temperature, rainfall, ground moisture and humidity can be used to construct static risk maps in the absence of interventions [54,55], and the impact of seasonality has been examined with careful mathematical modelling [56]. The present simulations demonstrate the impact of dry-season rainfall, vector refugia, and the mix of species on seasonal population dynamics. In the Garki weather data, no rainfall was recorded in the data for over six months. In a rainfall-driven model, drought of this depth and duration induces local extinction of the *An*. *gambiae sl*. populations. As a result, a baseline precipitation level of 1 mm per two weeks was artificially added to the data. In reality, this could be provided by low levels of rain that would not register in a daily rain measurement or by semi-permanent standing-water sources. Alternatively, there may be local fadeouts of the vector population requiring influx from refugia at the start of the rainy season when larger areas of habitat will become available. In this case, such refugia become very important in near-elimination conditions. The mix of species has strong effects on the seasonality as well. *An*. *funestus* is modelled with a slow integration of rainfall, with populations peaking at the end of the rainy season and decaying slowing into the dry season. This provides a low level of transmission in the first part of the dry season and has a substantial effect on the time-trough in parasitaemia. A combination of these factors in the present model can be used to explain both the lack of a strong seasonal oscillation in Namawala parasitaemia and the much more pronounced oscillation observed for adults in the Garki district.

Other important factors when comparing model simulation outputs to field data include the diagnostic sensitivity for parasitaemia. Each day of the simulation, each *in silico* individual has a simulated blood smear test which finds any parasites in 0.1 μL of blood, corresponding to a sensitivity of 10 parasites/μL as described in the malariatherapy analyses and the Garki report [22]. These were specialized studies and are not characteristic of typical slide sensitivities. Reducing the sensitivity to more typical values for field microscopy, such as 50 parasites/μL, reduces the measured prevalence and increases the mean detected parasitaemia, because all detected parasite densities are at least at the sensitivity threshold.

Other sensitive and uncertain parameters concern human infectivity to mosquitoes, which depends on a variety of factors [11,12]. Such infectivity has been studied in previous models [37,57,58], and the present model can be used to understand the effect of the development of immunity to the asexual stages upon the degree of transmission. The effects of decreased gametocyte densities are balanced by those of decreases in the level of inflammatory cytokines, which have been shown to limit transmission success of gametocytes [46,59]. Results from new field studies will advance understanding of the roles of different components of the human population comprising the human transmission reservoir, e.g., when studied by age and symptom-structures.

## Conclusions

It is important to understand system sensitivities to parameters and model assumptions governing within-host and mosquito-borne transmission dynamics. Mechanistic models can aid in exploring the effects of assumptions and the weights of each alternative in open questions. The present model recreates baseline transmission intensity and transmission patterns. Modifications to local parasite population antigenic diversity can match changes in temporal dynamics by age and smoothed detected prevalence by age. The mechanistic framework of antigen-specific immune responses and parasite population antigenic diversity provides a simple and parsimonious explanation for observed prevalence trends.

Key areas for further investigation include factors related to human immunity and its development over the course of exposure [14,32,37] and the duration of infections [24,60]. The malariatherapy data comprise an important resource for understanding within-host dynamics and informing population-level models [14], but they are limited in the questions they can be used to answer. New data will be required to support development of a fuller understanding of the effects of parasite diversity upon population dynamics, and models can be exercised to demonstrate the importance of such studies. Understanding present levels of parasite diversity and how they change with transmission comprises a potentially powerful new synergy between modelling and field studies.

In summary, mathematical models can demonstrate the implications of basic parasitological research and vector ecosystematics for the design and operation of the global campaign for malaria eradication [7,61-63]. Levels of population immunity affect the ability to measure parasitaemia, to correctly estimate the scales of the human infectious reservoir, and to detect, contain and eliminate parasite reintroductions to previously-cleared areas. Modern computer-based mathematical modelling provides a rich set of opportunities to explore model spaces that were too expensive to work with even a decade ago, and thus models such as the present one will become increasingly useful tools to the broader malaria research community.

## Competing interests

The author declares that he has no competing interests.
